# Tetris in the Nervous System: What Principles of Neuronal Tiling Can Tell Us About How Glia Play the Game

**DOI:** 10.3389/fncel.2021.734938

**Published:** 2021-08-27

**Authors:** Dana F. DeSantis, Cody J. Smith

**Affiliations:** ^1^Center for Stem Cells and Regenerative Medicine, University of Notre Dame, Notre Dame, IN, United States; ^2^Department of Biological Sciences, University of Notre Dame, Notre Dame, IN, United States

**Keywords:** glia, neuron, tiling, spacing, self-avoidance

## Abstract

The precise organization and arrangement of neural cells is essential for nervous system functionality. Cellular tiling is an evolutionarily conserved phenomenon that organizes neural cells, ensuring non-redundant coverage of receptive fields in the nervous system. First recorded in the drawings of Ramon y Cajal more than a century ago, we now have extensive knowledge of the biochemical and molecular mechanisms that mediate tiling of neurons. The advent of live imaging techniques in both invertebrate and vertebrate model organisms has enhanced our understanding of these processes. Despite advancements in our understanding of neuronal tiling, we know relatively little about how glia, an essential non-neuronal component of the nervous system, tile and contribute to the overall spatial arrangement of the nervous system. Here, we discuss lessons learned from neurons and apply them to potential mechanisms that glial cells may use to tile, including cell diversity, contact-dependent repulsion, and chemical signaling. We also discuss open questions in the field of tiling and what new technologies need to be developed in order to better understand glial tiling.

## Review

**FIGURE 1:**
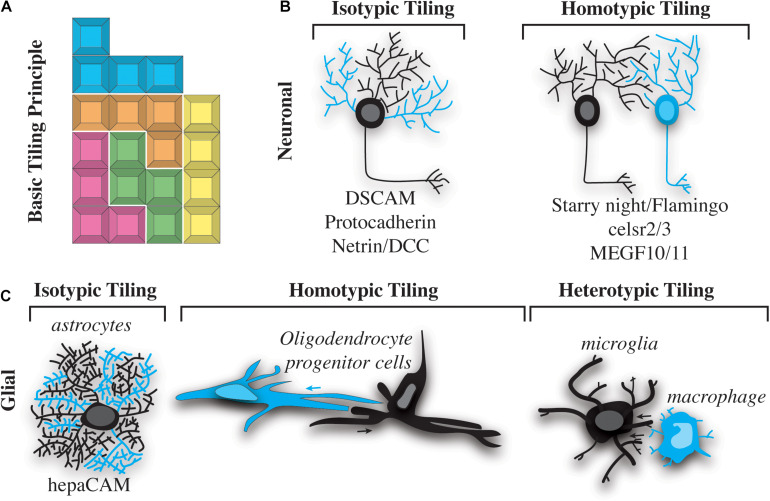
Types of nervous system tiling. **(A)** “Tetris” tiling principle. Different colored blocks represent cells of different types. Different blocks fit together, but do not overlap with one another. **(B)** Two basic types of neuronal tiling. Isotypic tiling occurs when sister dendrites of the same neuron avoid one another. Cyan dendrites do not overlap with black on the same neuron (left). Homotypic tiling occurs when two neurons of the same type (one shown in black and the other in cyan) avoid one another and their black dendrites do not overlap (right). A selection of molecules that facilitate these types of tiling are listed below the illustrations. **(C)** Three basic types of tiling occur in glial cells. Isotypic tiling occurs in astrocytes when branches of the same cell (cyan vs. black) avoid one another. hepaCAM is involved in astrocytic typing (left). Oligodendrocyte progenitor cells (OPCs) exhibit homotypic tiling whereby OPCs of the same type (cyan vs. black) avoid one another. The direction of this avoidance is shown by cyan and black arrows (center). Heterotypic tiling occurs when two different types of glial cells avoid one another. In this case a black microglial cell is shown avoiding a cyan macrophage (right).

Cells in the nervous system are precisely organized to ensure functionality of the body. Two universal principles have governed the assembly of such organizations. First, neural cells are over-produced during development and systematically eliminated to produce a mature organization. Second, neural cells non-redundantly organize in the nervous system to ensure efficient coverage of their respective areas. With these general principles, a collective body of literature demonstrates that distinct neural populations tile with themselves, resulting in a Tetris-like pattern of similar cells (Figure 1A). We know this evolutionarily conserved phenomenon has been observed from simple invertebrate nervous systems like those of *C. elegans* and *Drosophila* to the complex nervous systems of vertebrates such as zebrafish and mouse (Grueber et al., 2002; Grueber and Sagasti, 2010; Sundararajan et al., 2019). Both the peripheral and central nervous systems have distinct neural tiling events. Below, we discuss what is known about neuronal tiling and postulate ways in which some neuronal tiling concepts may be relevant to glial tiling. Instead of an exhaustive review of the current literature (Grueber and Sagasti, 2010; Lawrence Zipursky and Grueber, 2013; Sundararajan et al., 2019), we focus on specific concepts of neuronal tiling, together with what we know of glial cells, to highlight specific challenges the glial biology field faces in understanding glial tiling.

## Tiling and Cell Diversity

Tiling has been hypothesized to generate a non-redundant organization of neural cells. This definition of tiling implies that cells that tile exhibit distinct characteristics from cells that they do not tile with. It is widely accepted that neurons display such cellular diversity (Cajal, 1911; García-López et al., 2010). For example, sensory neurons across evolution display striking tiling of neurons with similar characteristics (class) but not those with non-similar features. This has been extensively studied in *Drosophila* where peripheral sensory neurons extend dendritic branches that cover the animal’s epidermis in a non-redundant manner (Grueber et al., 2002). These *Drosophila* sensory neurons are characterized in four classes depending on the dendritic complexity; class 1 neurons have less branch complexity while class 4 neurons display striking tree-like morphologies (Grueber et al., 2002). *C. elegans* similarly display sensory neurons that tile the dermis (Sulston et al., 1983; Smith et al., 2010; Albeg et al., 2011). In *C. elegans*, the PVD and FLP neurons have complex dendritic structures, while light-touch neurons like ALM and AVM have simpler morphology (Smith et al., 2010, 2013; Albeg et al., 2011). The extensive dendritic patterning of PVD and FLP demonstrate that these cells tile with one another but seemingly ignore ALM and AVM neurons, again supporting the hypothesis that like tiles with like (Smith et al., 2010, 2012, 2013). Further supporting this concept, converting AVM or other neurons into a PVD-like neuron causes new tiling between AVM and PVD (Smith et al., 2013; Yip and Heiman, 2016).

While it is generally accepted that such tiling occurs between “like” cells, this principle requires an in-depth understanding of “like.” Whereas our understanding of neuronal diversity is extensive, glial diversity is far less understood. Ramon y Cajal famously documented the extreme neuronal diversity that is present in the nervous system, but his drawings did not portray extensive glial diversity (Cajal, 1911; García-López et al., 2010). Del Rio Hortega, Ramon y Cajal’s protégé, identified many morphologically distinct non-neuronal cells (Río-Hortega, 1918). His documentation of macroglia (astrocytes and oligodendrocytes) and microglia has remained in our common vocabulary today (Bereciartu, 2020). However, even 100 years later, our understanding of the functional, morphological, and transcriptional diversity within subclasses of glia is immature, at least compared to our understanding of neurons. Thus to understand tiling, the glial biology field must gain a deeper understanding of diversity within glial populations.

At this point, we know that astrocytes display morphological diversity across several model systems and humans. In *Drosophila*, astrocytes associate with neurons, vasculature, and synapses and are required for circuit formation and neural function (Freeman, 2015). Astrocytes in the CNS form strict boundaries with neighboring astrocytes, suggesting they have strict tiling features. Supporting this, focal ablation of an astrocyte causes the neighboring astrocyte to fill its receptive area (Stork et al., 2014). This mirrors tiling experiments in neurons, showing that surrounding “like” cells sense the open receptive area and fill it. Astrocytes are also present in the vertebrate retina, particularly in the retinal nerve fiber layer where they tile to form a monolayer that facilitates the development of retinal vasculature (Panneer Selvam et al., 2018).

In vertebrates, astrocytes have distinct tree-like structures (Allen and Lyons, 2018). We know that the spinal cord in mice exhibits at least three morphologically distinct astrocytes including those that make the glial limitans, the fibrous astrocytes in the gray area, and astrocytes that compose the blood brain barrier (Molofsky and Deneen, 2015; Allen and Lyons, 2018). Zebrafish also have fibrous astrocytes in the spinal cord and astrocyte-like radial glia that produce the glial limitans (Bernardos and Raymond, 2006; Chen et al., 2020). Whether these morphologically distinct astroglial cell populations tile is not known.

The most outward-facing component of the CNS, the vertebrate eye, exhibits extensive tiling of both neuronal cells and glial subtypes. In addition to the astrocytes discussed above, the neural retina is tiled by a specialized glial subtype known as Müller glia. In general, Müller glia do not exhibit extensive morphological diversity, but mosaic labeling experiments utilizing BRAINBOW in mice and transgenic labeling in zebrafish have shown that they tile the retina with minimal overlap (Williams et al., 2010; Wang et al., 2017). Laser ablation of zebrafish Müller glia results in tiling gaps, which are filled in by processes of neighboring Müller in an example of homotypic tiling (Williams et al., 2010).

Despite the obvious diversity of glial cells in the nervous system, the number of currently defined glial subpopulations is at least an order of magnitude less than what is defined by neurons. Taking into consideration only a subset of neuronal cells such as excitatory neurons, the sheer amount of morphological diversity in distinct neurons is massive compared to known subsets of glial cells. It is of course possible that glial cells do not display such morphological diversity. However, it is also possible that the field has not yet produced a true catalog of diversity among glial populations, a task that will likely be required for a more complete understanding of which glia tile and the extent of glial tiling.

While it seems possible that glia are not as diverse as neurons and thus may not exhibit the same degree of tiling, recent advances in single-cell RNA (scRNA) sequencing demonstrate that glial cells are diverse, at least at the transcriptional level. Pioneering scRNA sequencing work in the mouse brain identified at least thirteen transcriptionally distinct oligodendrocyte populations in the adult brain (Marques et al., 2016). Since then, astrocytes, microglia, and Schwann cells have all been shown to exhibit transcriptionally distinct subsets of cells. For example, microglia in the developing brain can be subdivided into thirteen subgroups (Hammond et al., 2018). Collectively, these studies demonstrate impressive diversity among glial cells. However, the community is missing a link between transcriptional diversity and morphology. Recent approaches such as spatialomics could help to strengthen this link. But even spatialomic studies, as currently performed, lack the spatial resolution to dissect morphological diversity. Development of a system to image the morphological features of a cell and barcode those cells for later dissection and scRNA sequencing would facilitate cataloging the transcriptional and morphological diversity of glia, thereby allowing us to systematically dissect whether these diverse glial subtypes exhibit tiling.

An additional barrier to understanding glial tiling is the inability to label populations of morphologically distinct glia. *Drosophila* researchers have utilized mosaic labeling like MARCM to dissect neuronal tiling (Lee and Luo, 1999, 2001). Similarly, the *Drosophila* community generated specific GAL/UAS reporter systems to label distinct neuronal and glial populations, allowing for the visualization of distinct classes of neural cells. The GAL/UAS system uses regulatory regions to drive GAL4 expression in subsets of cells and has been a robust system for decades (Fischer et al., 1988; Kakidani and Ptashne, 1988). Advancing our knowledge of specific regulatory regions that identify subsets of glia would aid in the investigation of glial tiling. With this approach, subsets of glia could be labeled and investigated. The field could leverage techniques like ATAC-seq in combination with scRNA sequencing to identify such regulatory regions. Researchers could also adopt MADM (Zong et al., 2005) to study glia in vertebrates, a system similar to MARCM, which is used in *Drosophila*, in order to label subsets of cells (Zong et al., 2005). BRAINBOW has been proposed as such a tool but has not yet been fully adopted by glial biologists (Livet et al., 2007). This is likely because the current regulatory regions that drive the BRAINBOW fluorescent proteins in glia are often expressed in mitotic cells, leading to clonal labeling of cells instead of random distinct labeling of subsets of glia. Combining MARCM or MARCM-like techniques with tools made from newly identified regulatory regions would certainly allow researchers to dissect tiling by labeling smaller subsets of glia. In order to understand glial tiling, we need to catalog the functional, morphological and transcriptional diversity of glia subsets. Below, we discuss what is known about neuronal tiling and how this knowledge can be leveraged for a greater understanding of glial diversity and tiling.

## Cellular Mechanisms That Drive Tiling

Tiling of neural cells can be categorized into isotypic, homotypic and heterotypic interactions (Figure 1). Neuronal self-avoidance, a form of isotypic tiling (Figure 1B), occurs in all organisms, from invertebrates such as *Drosophila* and *C. elegans* to more complex vertebrates like mouse and human (Grueber et al., 2002; Hughes et al., 2007; Matthews et al., 2007; Smith et al., 2010, 2012). In fact, this phenomenon has been recognized for more than a century, since the drawings of Ramon y Cajal showed minimal overlap between branches of dendritic arbors in the vertebrate nervous system (Cajal, 1911; García-López et al., 2010). Despite its apparent conserved roles in nervous system organization, most of what is known about self-avoidance comes from studies of invertebrate nervous systems. A simple example of self-avoidance was observed in leech embryonic C-cells. C-cells are found in body wall muscle and consist of a single soma with approximately 70 parallel processes. Injections of dye into these cells revealed that growth cones initially extended to a particular location, eventually sorting and aligning in parallel (Jellies and Kristan, 1991; Baker and Macagno, 2007). Ablation of single C-cells caused other processes to expand into the vacant space while maintaining parallel orientation.

Self-avoidance behavior, similar to that seen in the leech, has also been observed in more traditional model organisms such as *Drosophila* and *C. elegans*. In *Drosophila*, MARCM clonal analysis of dendritic arborization (da) neurons in the body wall demonstrated that these neurons could be categorized into distinct classes (Grueber et al., 2002). Neurons of the same type showed little overlap with themselves (isotypic) and one another (homotypic), and closely apposed dendritic arbors appeared to “repel” one another. Further work utilizing live imaging of eGFP-expressing class IV da neurons showed this phenomenon in real time (Williams, 2004; Han et al., 2012). Generation of GFP-expressing cells also allowed for cell ablation experiments which further showed that neurons within different classes possess different capacities to repel “like” dendrites. When cell bodies of class IV neurons were ablated, other class IV dendrites and neurons filled in the empty space. Furthermore, when cells were ablated near the midline, dendrites from both the dorsal and ventral sides invaded, but still repelled one another, suggesting that even cells that were not normally immediate neighbors exhibit tiling and self-avoidance behaviors (Grueber et al., 2003).

Live imaging in *C. elegans* has also revealed that the nociceptive PVD neurons exhibit tiling and self-avoidance behaviors. *C. elegans* have two PVD neurons, each of which grows lateral processes throughout the body wall muscles and extends toward the head and tail (Smith et al., 2010; Albeg et al., 2011). Transgenic PVD:GFP animals display complex, non-overlapping dendritic branching (Smith et al., 2010). Self-avoidance of PVD dendritic branches can be partially explained by a contact-dependent event that occurs during dendritic branch outgrowth. In timelapse imaging analysis, growing PVD branches contact each other before retracting to reveal the stereotypical gap between PVD tertiary branches (Smith et al., 2010). Similarly, sensory FLP neurons extend toward the head and display a dendritic architecture resembling that of the PVD neurons (Albeg et al., 2011; Smith et al., 2013). The dendritic arbors of FLP and PVD display minimal overlap, which suggests that both have nociceptive functions (Chatzigeorgiou et al., 2010; Albeg et al., 2011) and furthers the concept that neurons with related functions tile in order to ensure full coverage of their receptive areas.

As in the above invertebrate models, live imaging techniques have also advanced our understanding of tiling in some vertebrate organisms. The zebrafish is particularly amenable to such studies, as embryonic and larval animals are transparent, and transgenic lines make it possible to visualize developmental processes in real time. Larval zebrafish have a transient population of peripheral neurons known as Rohon-Beard cells, analogous to trigeminal neurons which are present in the head early in development (Sagasti et al., 2005). Rohon-Beard cells, together with the dorsal root ganglia (DRG) and trigeminal neurons, are responsible for responses to thermal and mechanical stimuli. The sensory:GFP transgene mosaically labels trigeminal and Rohon-Beard cells in the developing zebrafish (Sagasti et al., 2005). Importantly, this mosaic labeling is somewhat analogous to MARCM clones in *Drosophila*, allowing for visualization of dynamic interactions between individual cells. Live imaging of GFP-labeled Rohon-Beard cells demonstrated that growing dendritic arbors contact one another, but then repel, and dendritic arbors rarely cross one another (Sagasti et al., 2005). Such contact-dependent tiling events mimic those visualized in *C. elegans* and *Drosophila* (Smith et al., 2010). Laser ablation of a single trigeminal neuron on one side of a zebrafish results in crossing of dendritic arbors across the midline, a phenomenon not observed in untreated animals. Furthermore, ablation of trigeminal neurons results in aberrant innervation of Rohon-Beard neurons into the head region (Sagasti et al., 2005). Further supporting a contact-dependent aspect of self-avoidance, transgenic embryos possessing only single, isolated trigeminal neurons elaborate more extensive dendritic arbors than their wild type counterparts.

Just as zebrafish sensory neurons have been shown to exhibit tiling and self-avoidance, so too have some non-neuronal cells of the nervous system. For example, oligodendrocytes have been shown to non-redundantly space themselves in the zebrafish spinal cord (Figure 1C). Timelapse imaging of oligodendrocyte development demonstrates that migrating oligodendrocyte progenitor cells (OPCs) contact each other but then quickly migrate in opposite directions (Kirby et al., 2006). Similar to neuronal tiling, the processes of OPCs contact each other during this event. While the contact of the processes is similar, OPC cell bodies move with the processes to migrate away from the adjacent OPC, a phenomenon that is not described in cell bodies after neuronal branches contact surrounding branches. The ablation or removal of neighboring cells has been the definitive experiment to demonstrate contact-dependent tiling of neural cells. In populations where tiling exists, cells neighboring ablated cells fill the gap left by the ablation. Mirroring observations in neuronal ablations, zebrafish OPCs enter into the space of an ablated OPC (Kirby et al., 2006). This pioneering work in zebrafish demonstrated the capacity for contact-dependent tiling of glial cells. Since then, OPCs have been shown to also tile in the brain of mice via contact-dependent mechanisms (Hughes et al., 2013). Ablation of surrounding mouse OPC causes the neighboring cells to enter into the ablated space, clearly demonstrating a mammalian example of the phenomenon shown in zebrafish (Hughes et al., 2013). It is now widely accepted that OPCs exhibit contact-dependent tiling, demonstrating a homotypic tiling phenomenon (Figure 1C).

While tiling is observed between two “like” cells, as evidenced by OPC tiling and sensory neuron tiling, growing evidence suggests that two “sort of like” cells can also tile with contact-dependent mechanisms. Such a tiling event represents heterotypic tiling. For example, OPCs in the spinal cord utilize a contact-dependent mechanism to restrict themselves to the central nervous system (Smith et al., 2014). This heterotypic tiling phenomenon occurs at the motor exit point, where myelinating cells from the peripheral and central nervous system ensheathe their respective PNS and CNS domains (Smith et al., 2014). The spacing between CNS and PNS counterparts creates a unique node. This tiling is proposed to ensure axon potentials can travel from the CNS to the PNS uninterrupted. At the motor exit point in zebrafish, motor-exit-point (MEP) glia serve as gatekeepers to the peripheral nervous system in order to prevent OPC migration into the PNS. Timelapse imaging revealed that OPCs extended short “sampling” processes into the PNS that eventually contacted MEP glia to cause OPC migration away from the PNS (Smith et al., 2014). Mirroring other experimental designs to demonstrate tiling, ablation of the MEP glia resulted in OPC migration into the ablated space. Such migration resulted in OPCs populating the PNS (Smith et al., 2014). Interestingly, this contact-dependent mechanism is not reciprocal for MEP glia; instead, MEP glia are prevented from entering the CNS by radial glia (Smith et al., 2016). Thus the interaction between OPC and MEP glia represent a heterotypic non-reciprocal tiling event. A similar heterotypic interaction likely occurs between OPCs and cells derived from peripheral boundary cap cells (Bron et al., 2007). These types of interactions have not been investigated in neurons.

Heterogenous, non-reciprocal contact-dependent spacing has also been observed with microglia in the developing zebrafish. Timelapse imaging after injury of the dorsal root ganglia sensory neurons causes migrating microglia to contact other microglia and macrophages (Green et al., 2019). Tracking these interactions demonstrated that a microglial cell contacting another microglial cell results in migration in the opposite direction of the contact, implying that microglia exhibit some contact-dependent mechanism to properly space themselves (Green et al., 2019). This microglia-microglia contact is another example of homotypic interactions with both cells displaying reciprocal migration away from the contact event. Microglia that contact macrophages also migrate away from the contact site, consistent with the idea that microglia tile with macrophages (Figure 1C). As demonstrated with other cell types, ablation of macrophages results in microglia migration into the ablated space, consistent with the idea that microglia tile with macrophage-like cells. However, macrophages do not display the same response with contact from microglia. This microglia/macrophage type of interaction is similar to the behaviors exhibited by MEP glia and OPC, which results in one cell repelling another, but not in a reciprocal manner. Macrophages do not appear to repel themselves either, leaving open the possibility that macrophages are either not repelled by “like” cells or the repulsion is driven in a context-specific manner (Green et al., 2019).

It is now widely appreciated that glia, like neurons, display both homotypic and heterotypic tiling. We know that neurons also exhibit tiling of their own processes, known as self-avoidance. This isotypic interaction ensures a single neuron’s branches can non-redundantly cover a receptive area. Despite clear evidence that astrocytes, microglia, and oligodendrocytes have extensive, non-overlapping, cellular processes or branches, isotypic tiling of glial processes has largely been neglected. One example of isotypic tiling has been demonstrated in satellite glia, which ensheathe dorsal root ganglia sensory neurons. In a typical unit, one to two satellite glia will non-redundantly ensheathe the cell body of the neuron. Although this organization was described many years ago, the mechanism of how such ensheathment is completed remains mostly unknown (Hanani and Spray, 2020). We do know that satellite glia ensheathe neurons shortly after the genesis of the neuron, at least in zebrafish, even before the neurons have extended axons (Nichols et al., 2018). In zebrafish, a single glial cell can extend two processes that encircle the neuron. This process appears to result in the two processes meeting during a contact event, eventually resulting in the retraction of one of the processes. The collective contact-dependent movement of these two processes from the same cell ensures non-redundant ensheathment of the neuronal cell soma (Nichols et al., 2018). Whether other cells that extend large cellular processes exhibit similar isotypic tiling interactions will be a fascinating area for discovery in the future.

While it is clear tiling is present in numerous neural cell populations, we still do not know the extent to which glia tile, or the temporal limitation of that tiling. There is now strong evidence that OPCs display tiling features, but whether mature oligodendrocytes also retain this feature is yet to be determined. It seems likely that such tiling must persist into myelination stages for oligodendrocytes and Schwann cells, otherwise myelin sheaths would overlap. Such a phenotype would result in an axon ensheathed without a node of Ranvier. An absence of tiling at the oligodendrocyte stage may also cause wrapping at a single myelin sheath to be composed of two distinct oligodendrocyte cells, seemingly wrapping each other’s sheath. Such a phenotype would likely not be visible by light microscopy due to the spatial resolution that would be required to demonstrate two sheaths wrapping each other. Similarly, although microglia demonstrate tiling behavior in zebrafish larvae (Green et al., 2019), it is unclear whether tiling persists in mature microglia.

In vertebrates, mature microglia non-redundantly tile the brain and spinal cord, suggesting that a tiling mechanism persists later in development to pattern the nervous system. Recently a study in mice demonstrated that microglial turnover results in new microglia that fill the receptive area. In this context, the neighboring microglial cells did not appear to immediately infiltrate the newly open space. However, new microglia completely filled the space, resulting in a population of microglia that nearly completely filled the receptive space (Eyo et al., 2018). It is possible that the mature microglia do not extend to fill receptive space and only immature microglia demonstrate this feature. Astrocytes, perhaps even more morphologically complex than both microglia and oligodendrocytes, fill gaps in tissue with cellular processes (Stork et al., 2014; Allen and Lyons, 2018). We know from studies in *Drosophila* that neighboring astrocytes fill the space of an ablated astrocyte, again strongly supporting that contact-dependent mechanisms impact tiling of glia (Stork et al., 2014). However, whether this phenomenon spatially patterns all astrocytes or subsets of astrocytes remains an open question.

The dynamicity of tiling of glial cells is also poorly understood. Given our lack of knowledge of glial tiling, few studies have tracked the ability of single glial cells to tile across the cell or animal’s lifespan. Critical periods, which are brief windows in which neural circuits can be morphologically modified (Ackerman et al., 2021), may suggest that there could be limited dynamicity of certain neural tiling events. It is not clear whether tiling occurs over time, during specific age spans or developmental stages, or whether it is plastic as the body responds to different environments, hormonal, or chemical experiences. Furthermore, whether tiling changes after injury to potentially repair the nervous system is not known. Despite the near universal phenomenon of neural cell tiling, our limited understanding of the molecular signaling that drives such behaviors in non-neuronal cells may be a significant limitation to testing out the dynamicity, and other concepts of tiling. Understanding these mechanisms will be crucial in deciphering the roles of glial tiling in health and disease.

## Molecular Signals That Mediate Tiling

Just as self-avoidance and tiling were first observed in invertebrates, much of what we know about the molecular and signaling cascades governing these processes has come from studies of model organisms such as *Drosophila* and *C. elegans*. In *Drosophila* class I dendritic arborization (da) neurons, the Down syndrome cell adhesion molecule (dscam) gene is necessary and sufficient to promote contact-dependent dendritic repulsion (Hughes et al., 2007; Matthews et al., 2007; Millard and Zipursky, 2008). The Dscam locus encodes for a protein with more than 38,000 splice variants (Schmucker et al., 2000). Mutations that reduce the number of Dscam splice variants result in failure of dendritic branches to recognize and repel sister branches. Conversely, ectopic expression of the same Dscam isoform on dendrites of different cells promotes repulsion of dendrites that would normally overlap (Matthews et al., 2007). Although this mechanism is required for self-avoidance in *Drosophila*, the mouse Dscam locus does not encode for extensive splice variants, and mice mutant for Dscam do not necessarily exhibit self-avoidance defects (Zipursky and Sanes, 2010). A notable exception is the adult mouse retina, where OFF bipolar cells expand their dendritic fields in the absence of Dscam (Fuerst et al., 2009). Therefore, while Dscam can mediate repulsion between dendritic branches, it is unlikely to promote self-avoidance with the striking diversity it does in *Drosophila*.

*Drosophila* Turtle (Tutl) is a conserved transmembrane member of the immunoglobulin (Ig) superfamily. In class I da neurons, Tutl is necessary for the prevention of growth of dendritic arbors in neurons with simple arbors and for self-avoidance of arbors in neurons with more complex dendrites (Long et al., 2009). Loss of the cytoplasmic face of Tutl does not result in self-avoidance defects, suggesting that Tutl acts as an extracellular ligand or receptor for another molecule to promote self-avoidance and limit dendritic branching (Long et al., 2009). Although Tutl has orthologs in mice and humans, null mutant mice display normal dendritic arborization, possibly due to redundancy with other molecules (Mishra et al., 2008). Therefore, like Dscam, Tutl may not represent a universal mechanism for contact-dependent self-avoidance in neurons across evolution.

Live imaging in *C. elegans* has revealed that PVD nociceptive neurons display dynamic dendritic branching during development and that overlapping of sister branches is prevented by a contact-dependent mechanism, as discussed above. Tertiary branches of PVD neurons grow until they contact another tertiary branch (Smith et al., 2010). Live imaging of these processes suggests that diffusible cues can promote dendritic self-avoidance. Loss of the *C. elegans* homolog of the axon guidance cue Netrin (UNC-6) results in a failure of self-avoidance during the period when tertiary branches develop (Smith et al., 2012). In this mechanism, the *C. elegans* homolog of DCC, the UNC-40 receptor, sequesters UNC-6 at the tips of dendrites. The interaction between sequestered UNC-6 and UNC-5 on apposing dendrites results in dendritic repulsion (Smith et al., 2012). This model of “catch and present” has been proposed in the mouse brain with Slit/Robo molecules (Gibson et al., 2014).

Although self-avoidance and neuronal tiling both promote a “like-repels-like” cellular organization, the molecular mechanisms governing these phenomena are not identical. As discussed above, Dscam promotes dendritic self-avoidance, primarily in invertebrates. Protocadherins are more likely to serve the role of DSCAM in vertebrates, as they display diversity in the number of isoforms expressed (at least 58 variants are encoded for by the genomic locus) (Garrett and Burgess, 2011; Lefebvre et al., 2012; Lawrence Zipursky and Grueber, 2013). Interestingly, other cadherin proteins are involved in self-avoidance mechanisms. Starry night (also known as Flamingo; fmi) encodes a seven-pass transmembrane cadherin that promotes self-avoidance at early stages of development and facilitates neuronal tiling at later stages by preventing dendritic overlap between neurons (Gao et al., 1999, 2000; Grueber et al., 2002; Sweeney et al., 2002; Matsubara et al., 2011; Kim et al., 2012). Whereas the function of DSCAM is not extensively conserved in mammals, cadherins function in both self-avoidance and tiling. Celsr2 and Celsr3 are two seven-pass transmembrane cadherin proteins evolutionarily related to Starry night/Fmi. Loss of these proteins in cultured rat neurons results in aberrant self-avoidance (Shima et al., 2007), indicating that this is not only a mechanism employed by invertebrate animals. Mosaic tiling in the mammalian eye is facilitated by MEGF10 and MEGF11, which ensure proper distribution of starburst amacrine and horizontal cells (Kay et al., 2012). Interestingly, this mechanism also ensures the proper distribution of retinal neurons.

Given that contact-dependent avoidance is common in tiling events, it is interesting to note that although several signaling and genetic mechanisms have been identified as promoting self-avoidance and tiling, mechanical cues governing these processes have not been identified. Given that mechanical cues have been shown to influence genetic and molecular signaling pathways, it will be important to investigate their roles in glial self-avoidance and tiling. For example, protocadherins play important roles in signaling pathways, but as members of the cadherin superfamily, they also have roles in adhesion. Adhesive forces influence signaling and vice versa, opening an intriguing area for study, both in neuronal self-avoidance and quite possibly in the same process in glial cells.

Despite progress on the cues that drive neuronal tiling, much less is known about the molecular signaling that ensures glial tiling. Recently, hepatocyte cell adhesion molecule (hepaCAM) has been shown to be required form non-overlapping territories of astrocytes in the mouse brain (Baldwin et al., 2021). hepaCAM is enriched in astrocytes and controls the competition for astrocyte territory in the developing mouse cortex. In this astrocyte tiling paradigm, both the intracellular and extracellular domain of hepaCAM are required for tiling, consistent with the hypothesis that extracellular interactions impact intracellular signals that alter astrocyte morphology. hepaCAM-induced tiling is dependent on expression-levels of hepaCAM between neighboring astrocytes. This paradigm introduces the intriguing possibility that the abundance of tiling molecules could alter the efficiency of neural cell tiling. Despite the identification of hepaCAM in astrocyte tiling, additional molecules that aid in tiling of other glial populations are mostly unknown.

## A Need for New Tools, Screens, and Functional Consequences

A major barrier to identifying molecular cues involved in tiling has been the lack of genetically tractable, stereotypical tiling events. The molecular cues that drive neuronal tiling were revealed in elegant experimental approaches in model systems like *Drosophila* and *C. elegans*. For years, most investigations of tiling molecules were performed in *Drosophila* where the peripheral sensory neurons were studied. The highly stereotypical morphology of those sensory neurons, combined with the genetic approaches widely utilized in *Drosophila*, revealed the molecules that dictate neuronal, or at least sensory neuronal, tiling (Grueber et al., 2002). The PVD neuron in *C. elegans*, with its similarly stereotypical morphology and tractable genetic system, introduced even more tiling molecules. Molecular dissection of glial tiling will likely require a high-throughput screening mechanism like that established in *C. elegans* and *Drosophila*. Screening of *Drosophila* astrocytes may reveal the molecules that govern astrocyte tiling. Similarly, *Drosophila* ensheathing glia, which mimic some features of myelinating glia, could reveal mechanisms of myelinating glia tiling. Other glia like microglia and oligodendrocytes will likely need to be investigated in vertebrate systems. Zebrafish could provide a powerful tool to reveal molecular cues of glial tiling. With the increased efficiency of CRISPR, genetic aberrations in a user-defined manner can be quickly and efficiently produced. Transgenic zebrafish have been created to label all the major glia subtypes including astrocytes, oligodendrocytes, microglia, and Schwann cells. The timing for the systematic screening of genes in zebrafish may be just right, as plentiful scRNA sequencing studies have produced lists of transcripts that are expressed in a given glial cell. Systematically perturbing these genes and scoring a noticeable glial tiling phenotype would likely identify new molecules used for cell tiling. Such approaches in *Drosophila*, *C. elegans*, and zebrafish could then provide a smaller subset of candidate molecules to investigate in mammals, which typically are not amenable to high throughput molecular discovery. An *in vitro* system, perhaps with induced cells from human iPS either from healthy or diseased individuals, could also provide a robust screening platform. As with any screening, the challenge is first identifying a robust phenotype that can reveal a lack of tiling. This will certainly require the development of new tools in the field.

Beyond the cellular and functional features that are left to be discovered, the field also has little understanding of the functional consequences of failed tiling. As a result, there is little knowledge of how changes in tiling could be presented in a clinical phenotype. One could speculate that in the absence of sensory neuron tiling, receptive areas would be double innervated by distinct neurons. This could in turn result in disruption of the spatial acuity of the sensory stimuli, causing two neurons to fire when usually only a single neuron responds. In astrocytes, the inability to tile may lead to astrocyte-free spaces in the brain, or, alternatively, redundant coverage of an area. Either scenario of disrupted astrocyte tiling could lead to a phenotype. Clinically, it has been shown that patients with peripheral neuropathies sometimes have spinal cells in the periphery (Coulpier et al., 2010). Such phenotypes in experimental cases are a consequence of failed tiling between CNS and PNS cells (Coulpier et al., 2010; Smith et al., 2014). However, it is only speculation that the lack of tiling resulted in neuropathy and not that the neuropathy somehow drove failed tiling. Perhaps the largest challenge to revealing a health consequence of failed tiling is the inability to experimentally disrupt tiling without inducing other phenotypes. Future research will be essential to identify a clear physiological consequence to failed tiling. Combining molecular screening with physiological readouts may further advance our knowledge of neural tiling.

## Do Glia and Neurons Cooperate to Ensure Tiling of Each Other?

The first molecules to govern neuronal tiling were cell-autonomously required in neurons. The prevailing model of neuronal tiling therefore focused on how proteins within a given cell ensure it can tile. However, these neural cells are present within packed nervous system tissue. Despite this, most models for neuronal tiling have focused on the intrinsic proteins that govern tiling. Recent evidence has challenged this cell-autonomous model. For example, the epidermis in *Drosophila* ensures dendritic tiling of sensory neurons (Han et al., 2012; Kim et al., 2012). Similarly, the dendritic array of the *C. elegans* PVD neuron is patterned by interactions with the epidermis (Salzberg et al., 2014; Dong et al., 2016). In such animals where this interaction is perturbed, self-avoidance (isotypic tiling) is also perturbed. Collectively, these studies demonstrate the non-cell autonomous role of surrounding cells to neuronal tiling. Considering glia were named because of their “glue” like features, seemingly tying the neurons together, it is logical that glial cells may play a prominent role in tiling of neurons. However, we know little about how glial cells could contribute to such neuronal tiling. Similarly, whether neurons have roles in glial tiling is not known. We do know, however, that glial cells quickly adjust cellular processes in response to neurons. For example, microglia extend cellular processes to contact neurons. Activity of the neurons has been proposed as one mechanism that controls microglial extensions (Li et al., 2012; Eyo et al., 2018). Astrocytes share a similar feature, extending toward neurons in an activity-dependent mechanism (Ackerman et al., 2021). Thus there is clear evidence that neurons can modulate glial processes; whether this also impacts tiling of glia will be an interesting avenue of study.

In a similar fashion, we know that glial subtypes can modulate other subtypes. For example, astrocytes and microglia reciprocally send molecular signals to each other to pattern the development of both cells and their response to disease environments (Liddelow et al., 2017; Gibson et al., 2019). We also know that microglia can clear debris of other cells. Such interactions could ensure proper tiling of one of the cells. This hypothesis is supported by the pruning of oligodendrocyte processes by microglia (Hughes and Appel, 2020), a mechanism that might ensure there is non-redundant coverage of oligodendrocyte sheathes. Microglia can phagocytose glial and neuronal debris (Wilton et al., 2019). Interestingly retinal astrocytes are initially overproduced and are eventually engulfed by microglia, which may be a newly identified mechanism of what promotes tiling (Puñal et al., 2019). Given that overextension or overproduction of branches/processes followed by retraction or pruning of those extensions is universally seen in tiling events, it seems possible, if not likely, that microglia could be central to many tiling events. Future studies that investigate the interaction of these subsets of glial cells with non-like glia could reveal more mechanisms that drive tiling of neural cells.

## Conclusion

Studies of neuronal tiling demonstrate that neurons of the same functional and morphological identity tile with themselves but not others. Neuronal branches also display self-avoidance, a type of isotypic tiling interaction. Although the extent of glial tiling is not known, there is evidence for homotypic, heterotypic and isotypic tiling in glia. Both glia and neurons appear to utilize contact-dependent mechanisms to drive neural cell tiling. Taking into account what we know about neuronal tiling, this suggests that glial tiling will likely be modulated by receptor-ligand pairs. Whether additional mechanical cues play a role in glial tiling is not known, but the contact-dependent feature of glial tiling may indicate that mechano-sensitive molecules play a role in glial tiling. Live imaging has been critical to our understanding of these mechanisms in neurons and will certainly be essential in investigating glial tiling, particularly since it allows for mechanistic investigation in the context of an entire animal. Additional tools need to be developed to define subpopulations of glia, particularly in vertebrates. This will allow us to form a more complete understanding of the repertoire of cellular interactions that shape the nervous system.

## Author Contributions

DD and CS contributed equally to the writing, editing, and figure construction of this manuscript. Both authors contributed to the article and approved the submitted version.

## Conflict of Interest

The authors declare that the research was conducted in the absence of any commercial or financial relationships that could be construed as a potential conflict of interest.

## Publisher’s Note

All claims expressed in this article are solely those of the authors and do not necessarily represent those of their affiliated organizations, or those of the publisher, the editors and the reviewers. Any product that may be evaluated in this article, or claim that may be made by its manufacturer, is not guaranteed or endorsed by the publisher.
